# *De novo* transcriptome assembly and quantification reveal differentially expressed genes between soft-seed and hard-seed pomegranate (*Punica granatum* L.)

**DOI:** 10.1371/journal.pone.0178809

**Published:** 2017-06-08

**Authors:** Hui Xue, Shangyin Cao, Haoxian Li, Jie Zhang, Juan Niu, Lina Chen, Fuhong Zhang, Diguang Zhao

**Affiliations:** Zhengzhou Fruit Research Institute, CAAS, Zhengzhou, China; Chinese Academy of Medical Sciences and Peking Union Medical College, CHINA

## Abstract

Pomegranate (*Punica granatum* L.) belongs to Punicaceae, and is valued for its social, ecological, economic, and aesthetic values, as well as more recently for its health benefits. The ‘Tunisia’ variety has softer seeds and big arils that are easily swallowed. It is a widely popular fruit; however, the molecular mechanisms of the formation of hard and soft seeds is not yet clear. We conducted a *de novo* assembly of the seed transcriptome in *P*. *granatum* L. and revealed differential gene expression between the soft-seed and hard-seed pomegranate varieties. A total of 35.1 Gb of data were acquired in this study, including 280,881,106 raw reads. Additionally, *de novo* transcriptome assembly generated 132,287 transcripts and 105,743 representative unigenes; approximately 13,805 unigenes (37.7%) were longer than 1,000 bp. Using bioinformatics annotation libraries, a total of 76,806 unigenes were annotated and, among the high-quality reads, 72.63% had at least one significant match to an existing gene model. Gene expression and differentially expressed genes were analyzed. The seed formation of the two pomegranate cultivars involves lignin biosynthesis and metabolism, including some genes encoding laccase and peroxidase, WRKY, MYB, and NAC transcription factors. In the hard-seed pomegranate, lignin-related genes and cellulose synthesis-related genes were highly expressed; in soft-seed pomegranates, expression of genes related to flavonoids and programmed cell death was slightly higher. We validated selection of the identified genes using qRT-PCR. This is the first transcriptome analysis of *P*. *granatum* L. This transcription sequencing greatly enriched the pomegranate molecular database, and the high-quality SSRs generated in this study will aid the gene cloning from pomegranate in the future. It provides important insights into the molecular mechanisms underlying the formation of soft seeds in pomegranate.

## Introduction

Pomegranate (*Punica granatum* L.) is an exotic deciduous tree in the Punicaceae family that has a long cultivation history in China. Because of its beautiful bright red, pink, and white colors, sweet and sour taste, and its recognition as a symbol for a fruitful and happy life, it is a favorite fruit among the Chinese [1]. Pomegranate is rich in nutrients such as vitamins [2], antioxidants [3], and others. It also can be a good complement to human nutritional requirements and has been thought to have anti-aging properties and to have potential use as cosmetic properties. Additionally, regular consumption of pomegranate also contributes to lowering blood pressure [4], cholesterol [5], and diabetes [6]. As something in pomegranate has the efficacy against bacteria and viruses, some skin diseases, and cancer [7–9], it can be used for clinical applications [10]. The earliest pomegranate varieties cultivated in China have hard seeds. The hard testa are not easy to swallow and thus, the nutrition is lost. The ‘Tunisia’ variety is a soft-seed pomegranate introduced to China in 1986 [11]. Its seed coat is relatively soft, edible, and easily swallowed, and its nutritional benefits can thus be easily obtained. After ten years of careful cultivation, ‘Tunisia’ has adapted to China’s environment. It is the best soft-seed pomegranate variety currently available.

In this study, the Illumina HisSeq 2500 *de novo* sequencing was used to analyze the differential gene expression between the soft-seed pomegranate ‘Tunisia’ and the hard-seed pomegranate ‘Sanbai’ during the development and ripening process. *De novo* transcriptome assembly is a method that supports the study of gene expression and transcriptional regulation at the RNA level [12]. RNA-seq is a newly developed high-throughput transcriptome sequencing technology that provides a new and effective method for large-scale transcriptome study. It can measure each transcript fragment sequence directly, detect single nucleotide differences, and there is no cross-activity as there is with the conventional gene chips. The high-throughput, high sensitivity, high resolution, properties allow RNA-seq to detect fewer rare transcripts and to directly analyze the transcriptome of any species [13]. RNA-seq data contribute to the development of SSR(Simple Sequence Repeats) and SNP(Single Nucleotide Polymorphisms) markers and have high versatility in related species, giving these markers important advantages over genetic mapping. Next-generation sequencing systems demonstrate great potential and are widely used in the discovery of new genes and SNP markers, identification of gene families, analysis of evolution, rendering of transcriptome map, determination of metabolic pathways, and soon [14].

We used this technology to identify the main genes expressed in the process of soft seed formation.

## Results

### Fruit growth status and seed size

**Fig 1:**
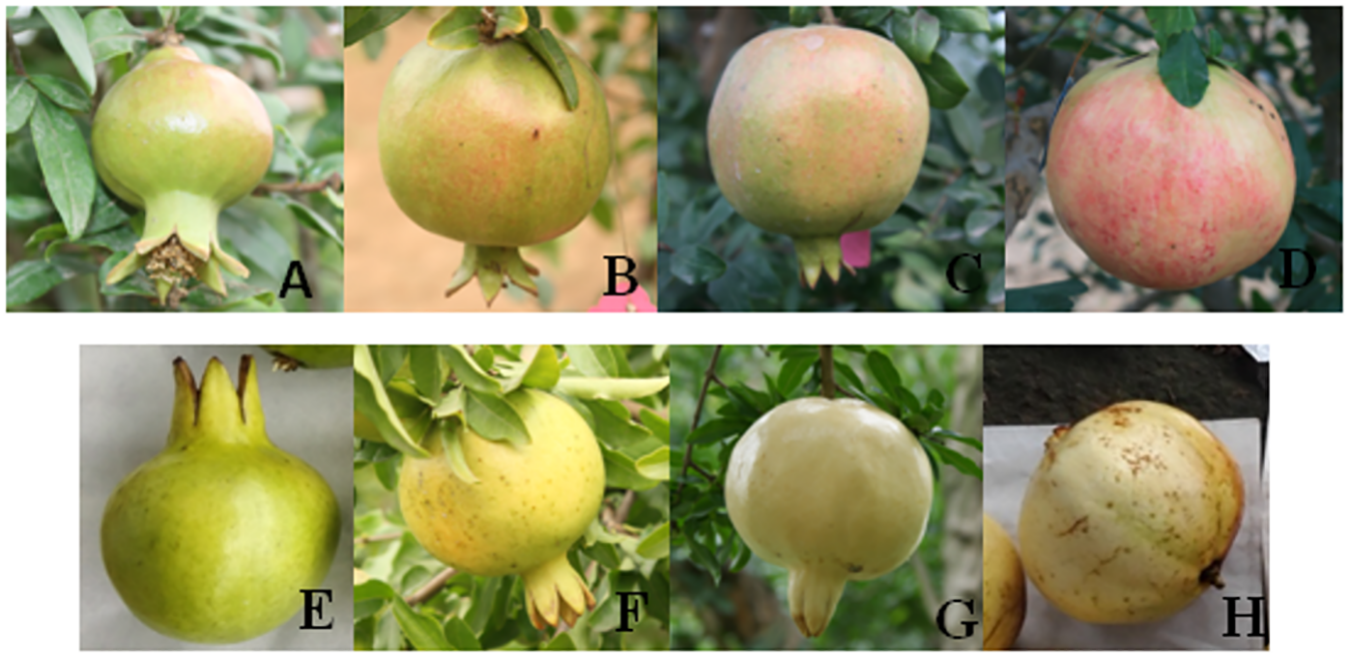
Fruit of two pomegranate varieties at different developmental stages. A, B, C, and D are ‘Tunisia’ at DAB 30, 60, 90, and 120, respectively; E, F, G, and H are ‘Sanbai’ at DAB 30, 60, 90, and 120, respectively.

**Fig 2:**
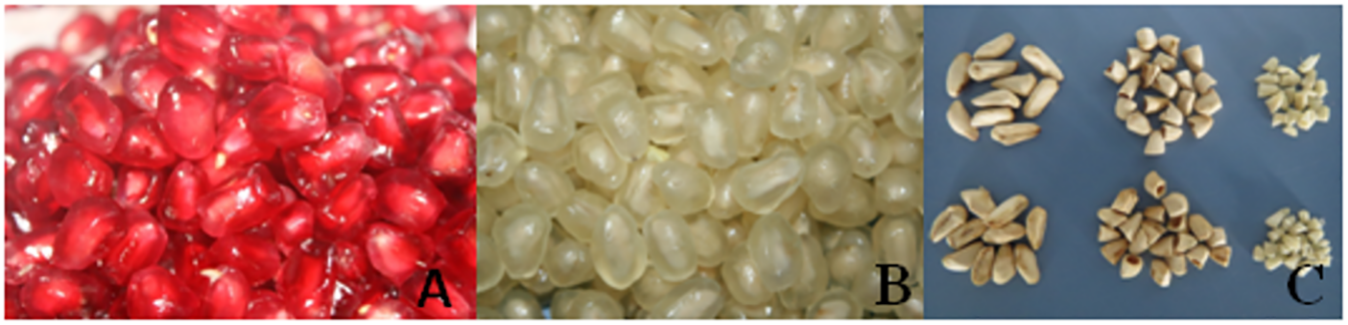
Arils and seed sizes of the fruit of two varieties of pomegranate. A and B are the arils of ‘Tunisia’ and ‘Sanbai’ (120DAB), respectively; C are the seeds of the two varieties: the first row is ‘Tunisia’ seeds, testa, and embryos, respectively; the second row is ‘Sanbai’, seeds, testa, and embryos, respectively.

Fruit growth status is shown in Fig 1. The appearance of the arils and seeds of the ‘Sanbai’ and ‘Tunisia’ pomegranate are shown in Fig 2. The data show that ‘Tunisia’ seeds were smaller and lighter in weight than ‘Sanbai’. As concluded from the weight measurements of the seed coat and embryos of the two pomegranate cultivars, respectively, the weight of the testa is the key factor that influences seed size.

### Seed hardness and lignin content

Seed hardness and lignin content were compared at the two different stages of the pomegranate varieties. Seed size, seed hardness, and lignin content at 30, 60, 90, and 120 DAB were tested. The seeds 30 DAB were not formed and could not be measured at this developmental stage. The results show that, overall, the hardness of the seeds in ‘Tunisia’ was different from that in ‘Sanbai’. The hardness of ‘Tunisia’ seeds decreased after 60 DAB and increased after 90 DAB; the seed hardness of the 90 DAB plants was the least, measured at 1.763 kg. While the seed hardness in ‘Sanbai’ decreased from DAB 60 to 120. The maximum value of seed hardness is 7.561 kg (Table 1).

**Table 1:** Seed-hardness of ‘Tunisia’ and ‘Sanbai’ at the different develop stage (kg).

Variety	30 DAB	60 DAB	90 DAB	120 DAB
‘Tunisia’	-	2.154^b^	1.763^b^	2.120^b^
‘Sanbai’	-	5.960^a^	6.814^a^	7.561^a^

Note: “-” at 30 DAB indicates that the testa and embryos had not yet developed. Letters represent significant difference, p < 0.05.

The results of seed lignin content in ‘Tunisia’ and ‘Sanbai’ show that the seed hardness and lignin content are correlated. The more lignin in seeds, the harder they are. (Table 2). From the SPSS(Statistic Package for Social Science) analysis, the content of lignin was positively correlated with the seed hardness, with a correlation coefficient of 0.946 (P < 0.01).

**Table 2:** Lignin content of ‘Tunisia’ and ‘Sanbai’ at the different develop stage (%).

Variety	30 DAB	60 DAB	90 DAB	120 DAB
‘Tunisia’	-	25.56^b^	23.86^b^	25.86^b^
‘Sanbai’	-	29.04^a^	29.42^a^	29.81^a^

Note: “-” at 30 DAB indicates that the testa and embryos had not developed. Letters represent significant difference, p < 0.05.

### *De novo* assembly and assessment of original transcriptome

**Fig 3:**
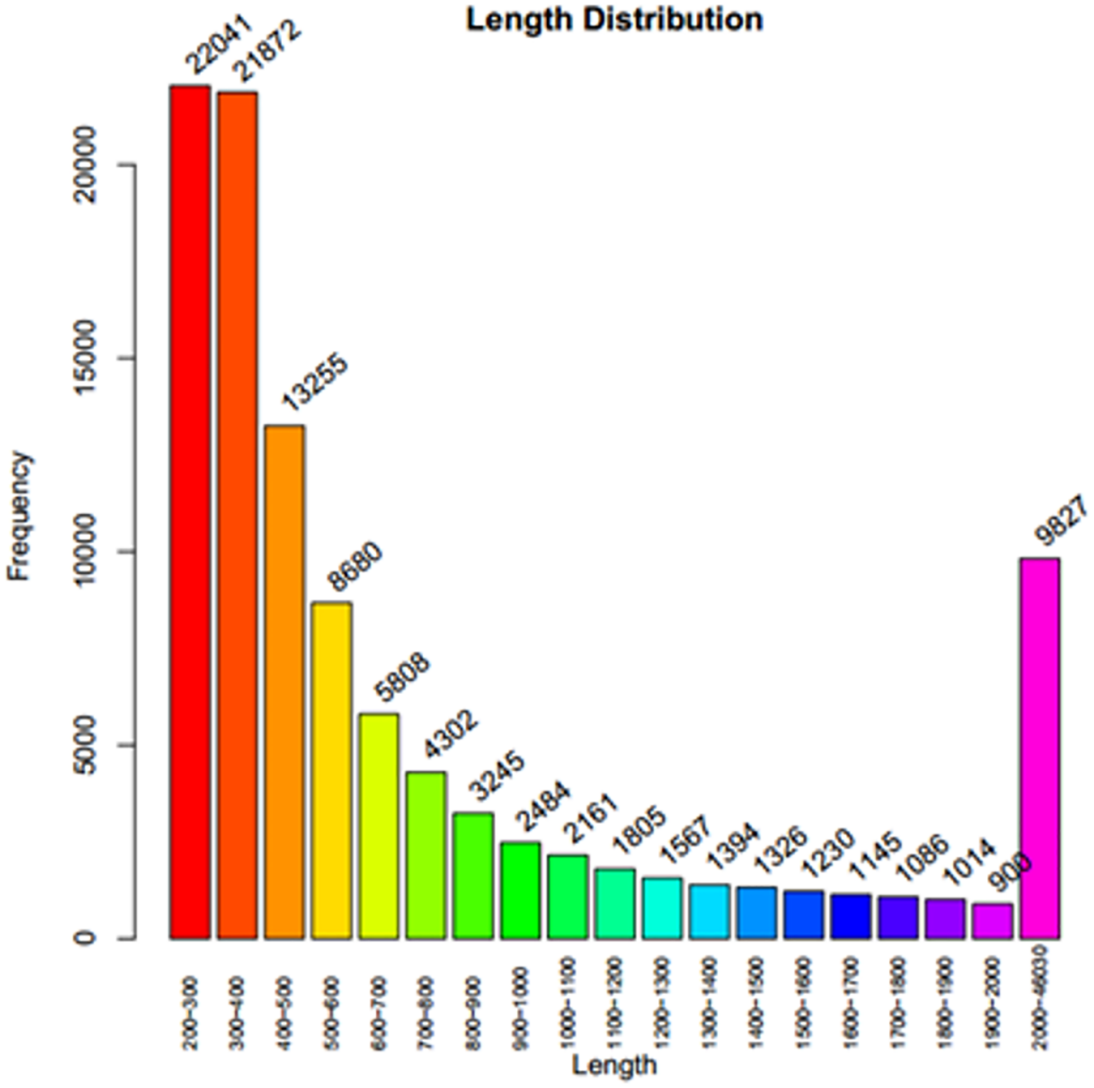
Length distribution of *Punica granatum* L. unigenes.

A total of 35.1-Gb of the original genomic data was sequenced at different developmental stages of ‘Sanbai’ and ‘Tunisia’(PRJNA371392). After filtering out the total data, over 200 million bp were filtered out. Q20(The probability of base error was 1%) reached more than 96%. The assembly sequence was sorted by length from biggest to smallest, accumulated in length when the sum was equal to 50% of the total length, the last of the fragment length was N50, which was 1,957 bp, and the average length of the contigs was 555 bp (Table 3). The transcription of the species was assembled and a total of 132,287 transcripts and 105,743 representative unigene sequences were obtained. The results of the assembly are shown in Table 4, and the distribution result shown in Fig 3.

**Table 3:** Length of *Punica granatum* L. unigenes.

Unigene Length(bp)	Total Number	Percentage (%)
200–400	43,913	42.17
400–600	21,935	21.06
600–800	10,110	9.71
800–1,000	5,729	5.50
1,000–1,500	8,253	7.92
1,500–2,000	4,375	4.2
2,000+	9,827	9.44
Total Number of Unigene	104,142	1

**Table 4:** Summary of unigene annotations of the assembled pomegranate (*Punica granatum* L.) transcriptome.

Annotated databases	Number of Unigenes	Percentage (%)
Annotated in CDD	33,132	31.5114
Annotated in KOG	36,756	34.9581
Annotated in NR	39,263	37.3425
Annotated in PFAM	38,882	36.9801
Annotated in Swissprot	30,573	29.0775
Annotated in GO	34,221	32.5471
Annotated in KEGG	9,011	8.5702
Annotated in at least one database	76,806	72.6346
Annotated in all databases	1,496	1.4228
Total unigenes	105,743	1

### Functional annotation

In order to understand the unigene sequence information, GO, KEGG, NR, Swiss-Prot, KOG, and other protein databases were used to compare the 105,743 unigenes from BLASTX. The results showed that 105,143 of unigenes had the same information, and 30,573 had specific protein function, which accounted for 29.08%, as shown in Table 4.

**Fig 4:**
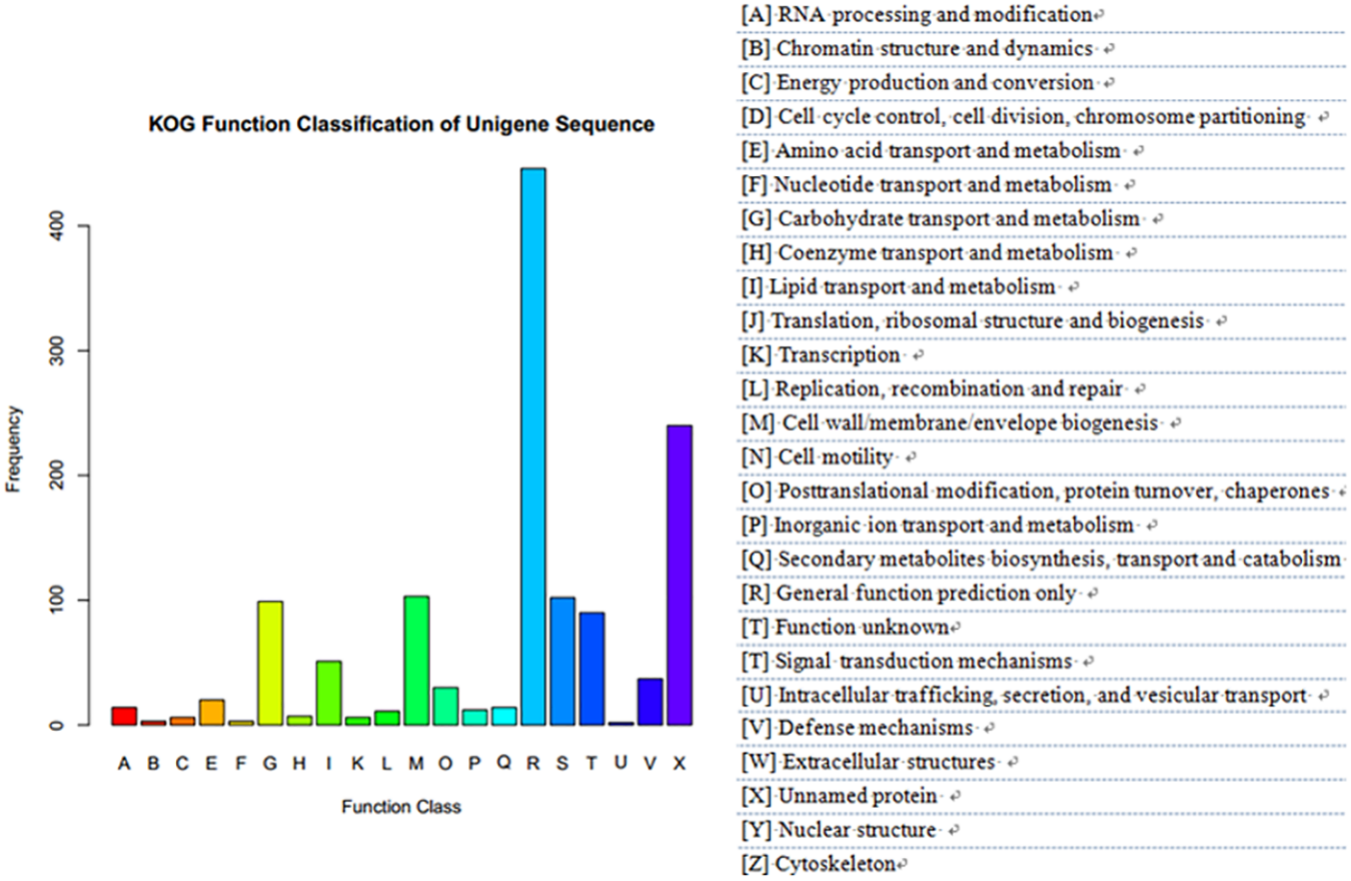
Number of pomegranate unigenes in each functional protein category (KOG).

In the functional classification system of KOG, we identified 36,756 unigenes with specific protein function definitions, which account for 34.96% of the total transcript and involved 20 KOG functional categories (Fig 4). Among them, the general function predictions were only for the longest reads; this was followed by secondary metabolite biosynthesis genes, transport and catabolic genes (11.34%), posttranslational modification, protein turnover, chaperones (10.93%), and energy production and conversion (10.12%) genes. In secondary metabolite biosynthesis group, there were genes involved in plant growth and development, which included amino acid transport and metabolism (3.64%), carbohydrate transport and metabolism (5.67%), lipid transport and metabolism (5.67%), inorganic ion transport and metabolism (6.48%), and other physiological and biochemical processes.

**Fig 5:**
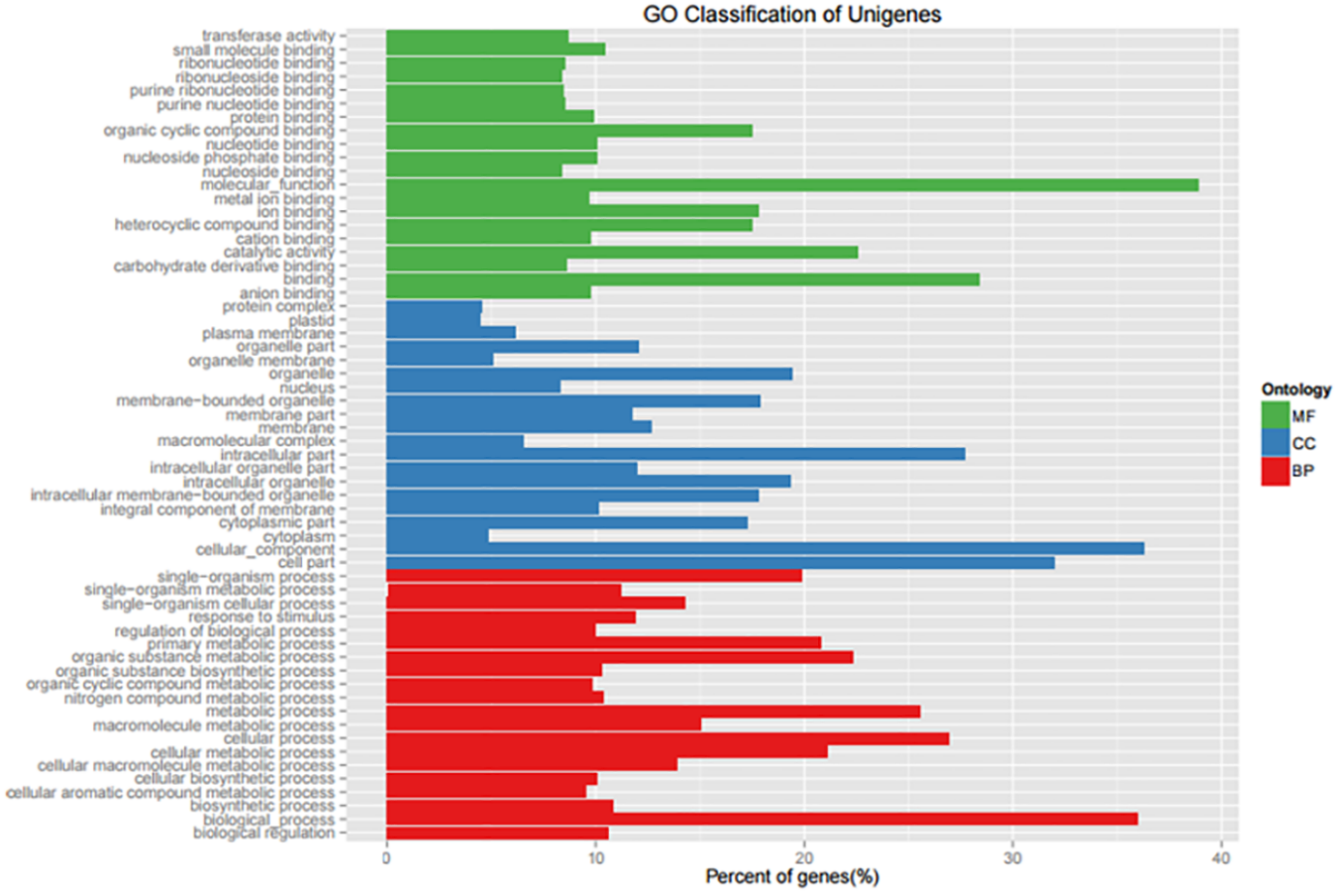
Number of pomegranate unigenes in each functional category (GO).

The Gene Ontology (GO) functional annotation classification system was used to compare the functions of 34,221 unigenes (E-value<1e^-0.5^). GO significant enrichment analysis identified genes that are mainly involved in cell composition (CC), molecular function (MF) and biological processes (BP) including 24, 19, and 16 functional categories as shown in Fig 5. In the cell components category, the genes differentially expressed were rich in cellular-component, cell part, organelle membrane, membrane-bound organelle, and macromolecular complex genes. In molecular function, the differentially expressed genes were rich in molecular function, binding, heterocyclic compound binding, iron binding, and organic cycle compound binding. In biological processes, the expressed genes were rich in biological process, cellular process, macromolecule metabolic process, primary metabolic process, and so on.

**Fig 6:**
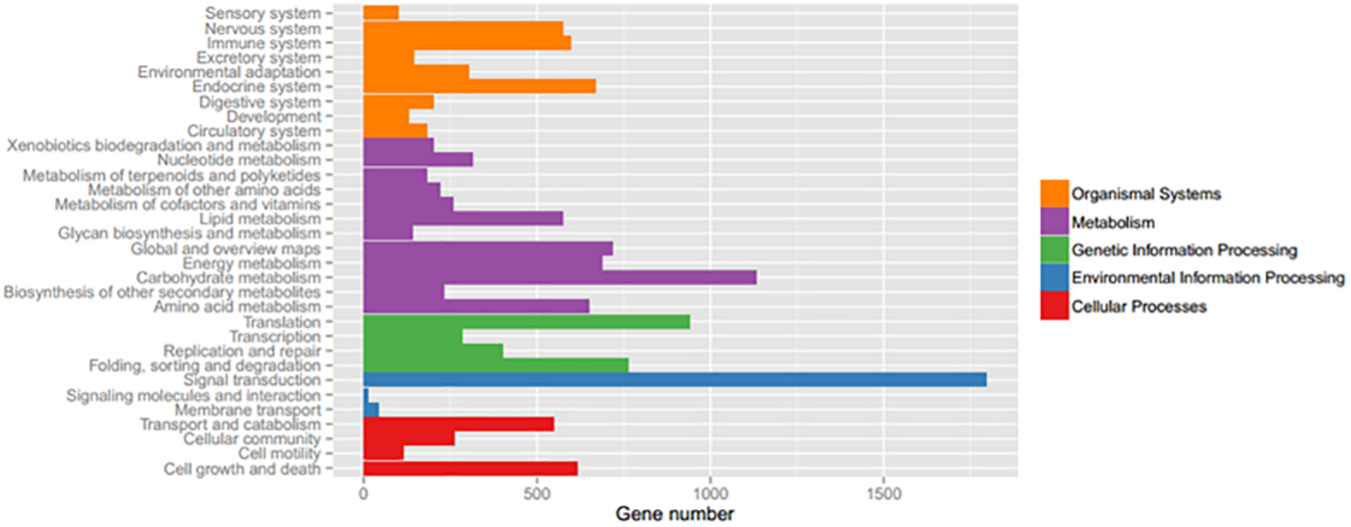
Number of pomegranate unigenes in each functional category (KEGG).

Based on the KEGG metabolic pathway enrichment analysis(Fig 6), it was determined that differentially expressed genes were involved in the following pathways: photosynthesis, benzene propane synthesis, phospholipid metabolism, ribosome metabolism, ubiquitin mediated proteolysis, and others.

### Differentially expressed genes

The differential gene expression between the two pomegranate varieties was analyzed with Cuffdiff software. Then, the number of up-regulated and down-regulated genes was counted. Finally, we determined the key differentially expressed genes between soft-seed and hard-seed pomegranate varieties through referencing the literature. The three periods of soft-seed variety were compared to each other: T1 vs. T2, T2 vs. T3, and T1 vs. T3. The three stages for the hard-seed variety were also compared to each other: S1 vs. S2, S2 vs. S3, and S1 vs. S3. Finally, the two varieties were compared in the three periods: T1 vs. S1, T2 vs. S2, and T3 vs. S3.

### Differential expression of genes related to seed hardness

From the analysis of all the differentially expressed genes, some genes related to cell wall formation were identified.123 different expression genes were found between T1 and S1,124 different expression genes were found between T2 and S2, 60 different expression genes were found between T3 and S3.

### Differentially expressed genes related to lignin biosynthesis

**Fig 7:**
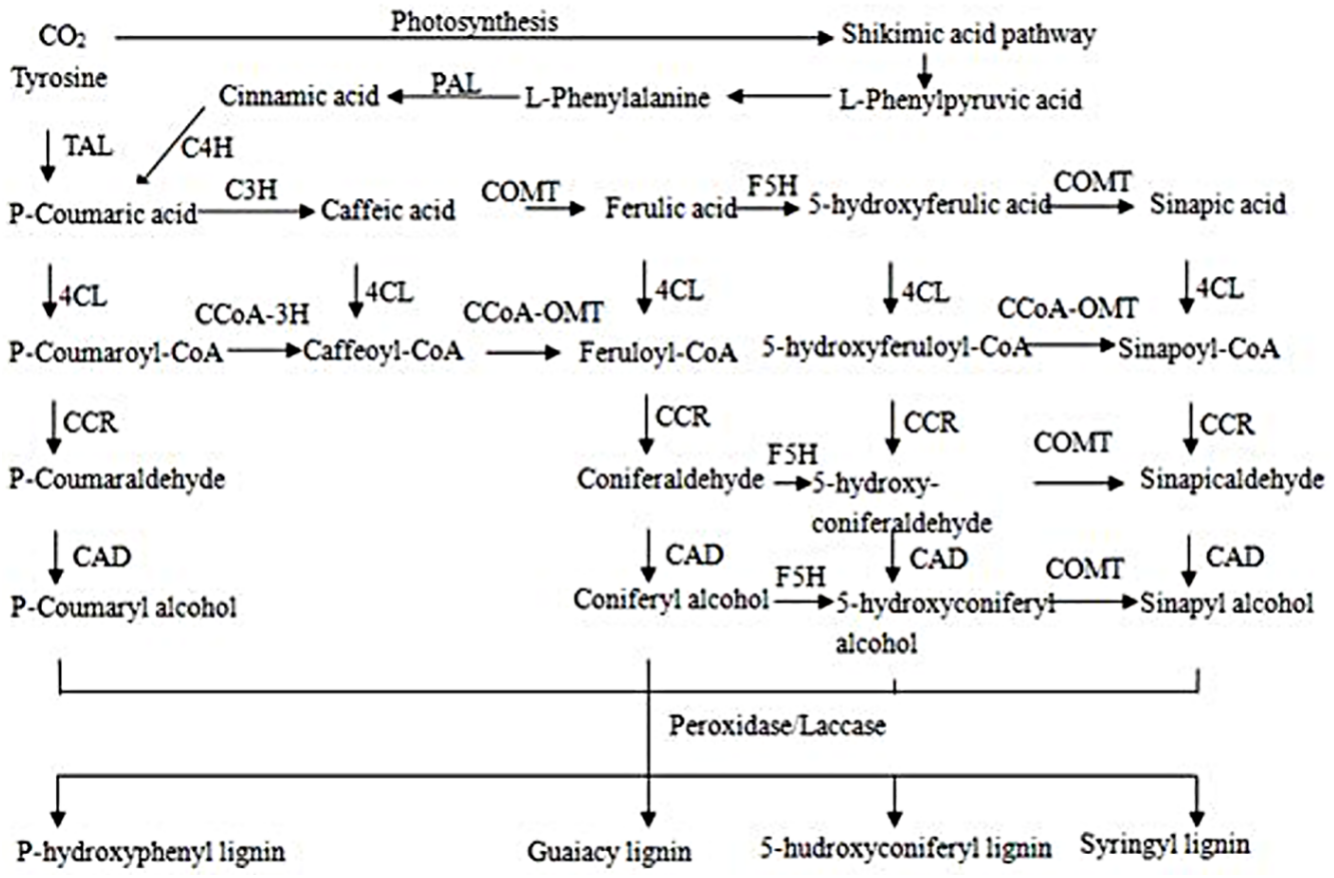
The lignin biosynthesis pathway.

In the growth and development of pomegranate, it was found that the seed hardness was related to lignin content, indicating that the key genes in the lignin biosynthesis pathway were also the key genes in the process of our differential gene selection(Fig 7)**[15]**. The differentially expressed genes related to lignin biosynthesis were compared between samples. We found differences in the gene expression at different stages of the same species and there were differences in the expression of different genes in the same stage.

Comparisons between T2 and S2 revealed that C4H (Cinnamate 4-hydroxylase), cinnamoyl coenzyme A reductase (Cinnamoyl CoA reductase), and PAL (phenylalanine ammonia lyase) gene expression was much higher in S2 than T2. The changes in lignin synthase gene expression at 60 DAB and 120 DAB were obviously different; most of the lignin biosynthesis genes in 60 DAB were up-regulated in S2. Among the lignin synthesis related genes expressed in S3 and T3, the expression of peroxidases 11, 15, and 64 was higher in T3 than S3. The expression of laccases 9, 12, and 14 was higher in S3 than that in T3. At 120 DAB, the expression of *CCR*, *PAL*, *CCoAOMT*, and *HCT* was up-regulated in S3. These proteins are considered to be key enzymes in the synthesis of H-lignin and G-lignin. The expression of *COMT* and *F5H*, which are the key enzymes in the synthesis of S-lignin were lower in both pomegranate varieties.

Our biological information analysis also indicated that some transcription factors also play an important role in lignin synthesis. We compared the two varieties in the same period and calculated the statistical number of transcription factors (Table 5). For example, in the T3 and S3 comparison, there were obvious differences in the expression of *9 MYB*, *14 NAC*, *12 WRKY*, *2 MYC*, and *6 bHLH* transcription factor genes. The expression of the *MYC* transcription factor in S1, S2, and S3 was higher than that in T1, T2, and T3. The expression of *WRKY31*, which belongs to WRKY transcription factor family, was higher in T3 than S3, while the expression level patterns of *WRKY56* were the opposite of those of *WRKY31*. The expression of *MYB305* in S3 was higher than that in T3.

**Table 5:** Differentially gene expression patterns of transcription factors related to lignin synthase.

DEG Set	MYB	NAC	WRKY	MYC	bHLH
T1 vs. S1	24	11	11	0	9
T2 vs. S2	25	11	13	1	12
T3 vs. S3	9	14	12	2	6

### Differential expression of other seed-hardness related genes

In addition to lignin synthesis related genes and transcription factor expression, the expression of flavonoid genes in different growth periods of the different pomegranates varieties was also different. Most of the expression of flavonoid related genes in T2 were higher than S2, such as flavonol synthase. At the same time, the expression of phenylalanine aminotransferases such as apotransaminase, was higher in T2 than S2. There were 17 cellulose related genes identified when we compared the different gene expression between S2 and T2, including 9 up-regulated genes in T2 and 8 up-regulated genes in S2. Among them, the expression of α-1,4- dextran protein synthesis, the cellulose synthase catalytic subunit 7, and the cellulose synthase catalytic subunit 8 were significantly up-regulated in S2. The expression of the genes related programmed cell death in T1, T2, and T3 was higher than that measured in S1, S2, and S3.

### Quantitative RT-PCR (qRT-PCR) analysis

**Fig 8:**
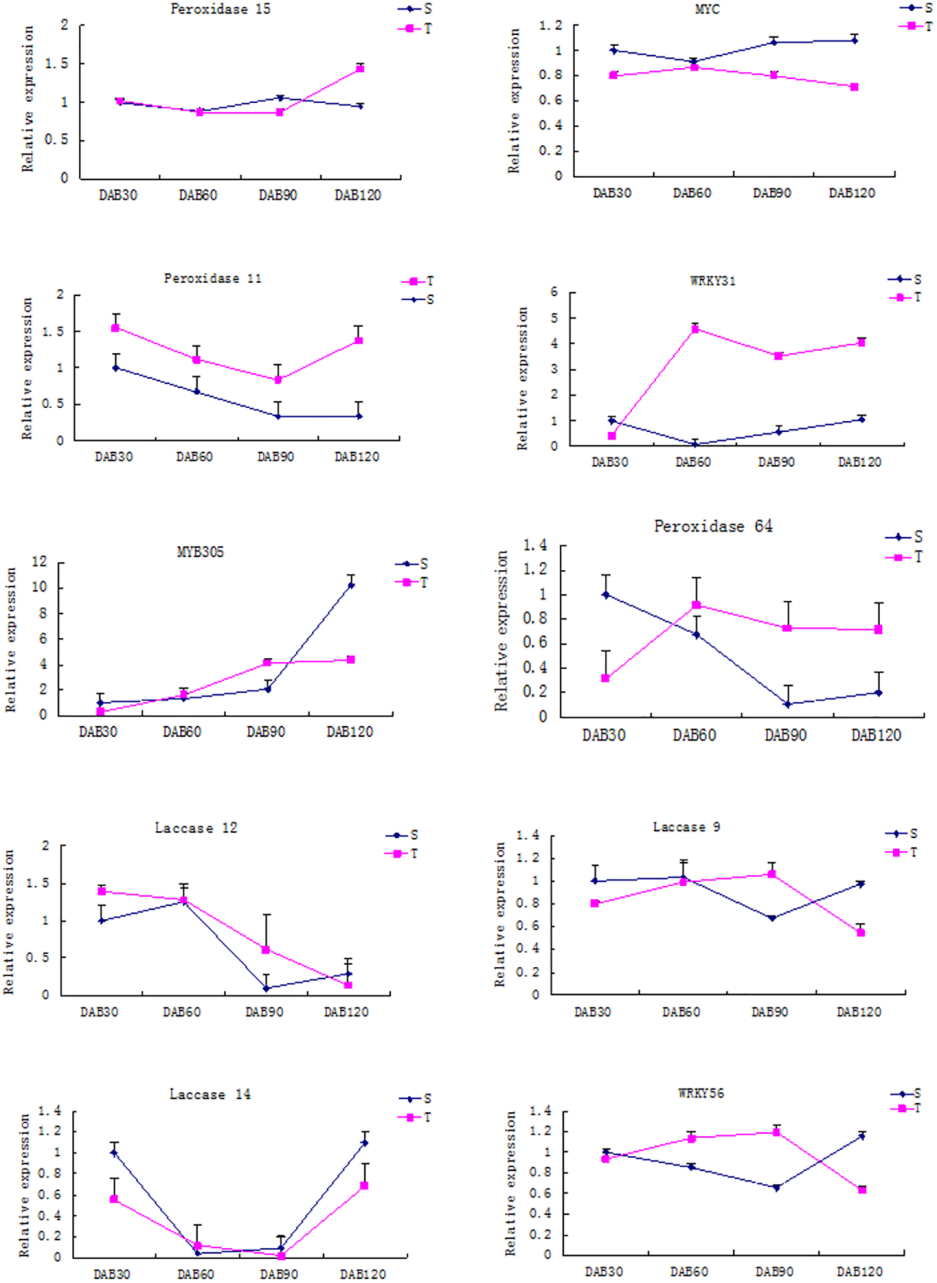
Differential gene expression analysis results analyzed via qRT-PCR. S: ‘Sanbai’; T: ‘Tunisia’.

Based on the above analysis and previous studies, as well as the experimental basis, we found several genes related to the formation and hardness of the seeds from the assessment of differential gene expression. According to the difference of gene expression, genes such as *CCR*, *CCoA-OMT*, *peroxidase*, *laccase*, *wood dextran*, *MYB*, *WRKY*, *MYC*, etc. showed significant changes in expression level. To confirm the reliability of the results from Illumina sequencing technology, 12 differentially expressed genes were selected for RT-PCR to analyze the expression of genes in different stages of seed development. These 12 genes were identified as the transcription factors WRKY16, WRKY56, WRKY31, MYB30, and MYC and the lignin-related genes *Laccase9*, *Laccase12*, *Laccase14*, and *Peroxidase11*, *Peroxidase15*, *Peroxidase42*, and *Peroxidase64*. The qRT-PCR results are summarized in Fig 8.

The expression levels of MYC transcription factors differed significantly between the two varieties. The expression of *MYB305* in ‘Tunisia’ was up-regulated at 60 and 90 DAB, and down-regulated at 30 and 120. DAB The expression of *MYB305* was the opposite of ‘Tunisia’ in the ‘Sanbai’ variety. Additionally, the expression of *WRKY56* in ‘Tunisia’ was up-regulated before 90 DAB and then decreased, which was the opposite of its expression pattern in ‘Sanbai’. The expression of *WRKY31* in ‘Tunisia’ first increased, decreased, and finally increased again, while in ‘Sanbai’, the expression at 60 DAB decreased and then increased. The expression of *WRKY16* in ‘Tunisia’ decreased after 60 DAB and then increased; in ‘Sanbai’ it decreased by 60 DAB, increased, and finally decreased again. The relative expression of *Laccase 9* was down-regulated at 90 DAF and up- regulated at 30, 60, and 120 DAF in ‘Sanbai’. The relative expression of *Laccase 12* was down-regulated at 30 and 90 DAF, but up- regulated at 120 DAF in ‘Sanbai’. The relative expression of *Laccase 14* was up-regulated at DAF 30, 90 and 120 in ‘Sanbai’. The relative expression of *Peroxidase 11* was down-regulated in all four flowering periods. The relative expression of *Peroxidase 15* was down-regulated at 120 DAF and up- regulated at 90 DAF in ‘Sanbai’. The relative expression of *Peroxidase 42* and *Peroxidase 64* was up- regulated at 30 DAF and down-regulated in the following three periods in ‘Sanbai’.

## Discussion

The understanding of the pomegranate on a molecular level is weaker than that for grape, peach, pear, and other fruit trees, and the genetic background is not well understood. In recent years, the Illumina sequencing technology based on the RNA-Seq platform has been widely used, especially in the species that have not yet been sequenced.

Studies on the pomegranate seed coat hardness have been conducted. Work from Dalimov et al. [16] indicated that the content of lignin and cellulose in the seed coat was the main component for seed hardness. Lignin is a natural component of secondary cell walls, and is involved in the formation of tubular cells, thick-walled cells, stone cells, and structural fibers [17]. This is consistent with our pomegranate experiment described here. SPSS correlation analysis showed that lignin and seed coat hardness are positively correlated with each other at a correlation coefficient of 0.946 (P<0.01). The lignin content in ‘Tunisia’ was lower than that in ‘Sanbai’. Han et al. [15] used transcriptome high-throughput sequencing to screen for differentially expressed genes in soft-core and hard-core hawthorns and showed that C4H, HCT, C3H, CCR, CCoA-OMT, F5H, and CAD are related to lignin biosynthesis; and that four NAC transcription factor encoding genes and 12 MYB transcription factor encoding genes were significantly down-regulated in the ‘Kaiyuanruanzi’ soft-core variety of hawthorn. Hu et al. [18] studied the comparative proteomics of the fruit and the inner skin of the peach at different developmental times. The difference between the inner fruit and the skin of the fruit was found to compete with the other proteins. According to the determination of the content of lignin in T2 transgenic lines, Liu et al. [19] found that the content of lignin in the transgenic lines increased by 22.47% compared to the control. The study of lignin degradation enzymes mainly focused on the enzyme system of white rot fungi, and the most important lignin degrading enzymes were lignin peroxidase, manganese dependent peroxidase, and laccase [20]. Lignin type and content are different in the different tissues of the same species and across different species.

In recent years, a large number of studies have shown that the regulation of gene transcription level may be one of the most critical regulatory points in the development of plant tissues [21]. The transcription factors that play a role in this process vary, and the NAC and MYB transcription factors are mainly regulated the ynthesis of lignin. Zhong et al. [22] found that NAC transcription factors were associated with the secondary cell wall thickening in tobacco fibers. The *PgCOMT* gene was cloned from the seed coat of the pomegranate and the relative expression in the seed coat of the pomegranate was consistent with that described in tobacco [23]. The transcription factors related to lignin biosynthesis were cloned and analyzed [24]. In addition, the PpNAC157, PpNAC105, PpNAC156, and PpNAC154 transcription factors can also significantly promote the synthesis of the secondary cell wall in poplar [25]. Studies show that the MYB transcription factors and the biosynthesis of lignin in the dicot wood[26]. Over-expression of *MYB46* and *MYB83* genes in tobacco can stimulate the expression of genes related to the biosynthesis of cellulose, xylanase, and lignin, and can lead to abnormal thickening of the secondary cell wall in some tissues as well as ectopic deposition of lignin [27]. In conclusion, the synthesis pathway of lignin is regulated by MYB and NAC transcription factors. This is also consistent with the results from this experiment, but the results of this experiment show that WRKY transcription factors also play a role in lignin regulation in pomegranate.

Testa formation may be related to the production of cellulose, hemicellulose, and callose in addition to lignin, biosynthesis. The transcriptome data show that genes involved in cellulose and xyloglucan synthesis significantly change in their expression. In short, in fruit trees, seed formation is a very complex biological process and to understand its molecular mechanism further study is needed.

## Conclusion

The Illumina HiSeq 2500 high throughput sequencing technology was used to sequence and analyze gene expression during the formation of seeds in different varieties and development status of pomegranate, and the overall gene expression during the formation of the pomegranate fruit seeds was revealed from original data of 35.1-Gb. The sequences of gene ORFs and the new SSR molecular markers were obtained. This study provides a rich data resource for the development of research on pomegranate growth, seed formation, SNP and SSR, and provides a theoretical basis for the cultivation of new varieties of soft seed fruit.

## Materials and methods

### Plant materials

Materials were collected from the Zhengzhou Fruit Research Institute, Chinese Academy of Agricultural Sciences, Xingyang, Henan. The average temperature in Xingyang city is 14.3°C, the average annual rainfall is 641.7 mm, the annual daylight is 2,367.7 h, and the annual sunshine percentage is 54% [28]. Bloom time was noted when 50% of the pomegranate flowers had opened. Seeds for the analysis of lignin deposition and lignin content and measurement of seed hardness were collected at 30, 60, 90, 120 days after blooming (DAB). At each collection time, 12 fruits were collected for each accession. Nine of these samples were flash frozen in liquid nitrogen and stored at -80°C for future RNA extraction. Using the TA-XT texture analyzer(Stable Micro System Co. Britain), we determined the seed hardness, and the measurement the lignin content of the seed was performed with a TU-1901 spectrophotometer(Beijing Pute Co. Beijing, China). All experiments were repeated three times.

### Total RNA extraction, cDNA library construction, and sequencing

RNA was extracted using the BioTeke general plant RNA Extraction Kit, and using RNA Nanodrop 1000 micro UV visible spectrophotometer(Thermo Scientific,) determined RNA purity and integrity after separation and purification. A cDNA library was constructed from the total RNA extracted for enrichment. The rRNA was removed and mRNA fragments were collected. Random primer synthesis was used to produce cDNA; PCR amplification was used to increase fragment length. After the construction of the cDNA library was completed, the Illumina HiSeq 2500 platform (Bustan biological company,Beijing,China) was used to carry out the sequencing of the genome.

### *De novo* assembly of transcriptome

We used the transcriptome splicing of the Trinity software [29] (version: v2014-07-17; parameter for the default parameters) for sequence assembly, which was divided into 3 parts: 1) Inchworm assembly where read sequences were used to construct the K-mer library; and according to the K-mer overlap, greedy K-mer extension could occur through the construction of contigs. 2) Chrysalis: contigs, Inchworm overlap, and K-mer-1 reads were supported based on clustering. The contig clustering can be clustered together for the construction of component deBruijn graphs, combined with the sequencing data for the comparison to deBruijn graphs. 3) Butterfly: according to the different components of the information from the Chrysalis gene transcription and the construction of the spliceosome, transcripts were sequenced. Finally, the unigene sequence data of the corresponding species, according to the component information, could be used to carry on the annotation and expression analysis.

### Open reading frame (ORF) prediction

Unigenes were predicted using open reading frame (ORF) software; the maximum was selected as the final ORF of the unigene, and the corresponding gene and protein sequences were obtained by Getorf [30]. Each unigene from the samples was predicted by the ORF; if a unigene was predicted to have multiple ORFs, the longest ORF was used to identify the transcription sequence. We translated each readable frame of the unigenes into an amino acid sequence, and determined the start of each fragment of the reading frame, the termination of the site, and its length and GC content.

### SSR analysis

Simple sequence repeat (SSR) markers are highly polymorphic, and have good repeatability and co-dominant inheritance. We used MISA [31] analysis and unigene software to obtain the SSR markers for pomegranate. The primers were designed using Primer3.0.

### Gene annotation

Using unigene software, BLAST [32] database comparison, NR[33], SwissProt [34], GO [35], KOG [36], and KEGG [37], we accessed the unigene annotation information. Blastx, NR, and Uniprot databases were used for homology comparison; an e-value ≤ 1 × 10^−5^ of most of the results from the screening was a function of the assembly of the unigenes. Blastx and COG (KOG) databases were used for homology comparison; the e-value ≤ 1 × 10^−5^ of most of the results of the screening was a function of the assembly of the unigene homology.The GO Unigene (https://www.blast2go.com/) software was used to annotate the GO, NR, and to define the distribution of unigenes. In living organisms, different genes are coordinated to perform their biological functions, and the function of the gene based on Pathway analysis is helpful for further understanding gene function. KEGG is the main public database of pathway. The KEGG was annotated using Kobas (V2.0).

### Analysis of differential gene expression

Bowtie (V1.0.0) software was used to analyze the expression of mapped genes, and the expression from gene reads was done on the order of the whole genome. The expression of the genes was calculated using Reads Per Kb Million (RPKM) [38].

### Quantitative RT-PCR (qRT-PCR) analysis

The qRT-PCR method was previously described by Feng Hu et al. [39]. The real-time fluorescence quantitative instrument was Roche 480, and the fluorescence quantitative kit from Biotake of SYBR Real-time PCR Premixture. The primers shown in Table 6. The reactions were prepared as instructed by the SYBR Real-time PCR kit. Briefly, the reaction included 10 μl 2 × Premixture, 2 μl of each primer, 2 μl cDNA template, and 4 μl double distilled water. The reactions were repeated three times for each sample set. The reaction program was as follows: an initial 95°C denaturation for 5 min; followed by 95°C denaturation 20 s, 60°C annealing 20 s, 72°C extension 20 s, for 45 cycles. Data were analyzed using relative quantitative analysis, and 2^-△△CT^ to analyze differences in the relative expression of genes.

**Table 6:** Primers for real-time and quantitative real-time PCR.

Primer name	Primer sequence 5′-3′
WRKY31F	ATGTCCGACCACCACCAAGC
WRKY31R	CTGCAAGAGCAGCGGTGAAG
WAKY56F	TACTACCTCTAGCCCTAGATCTCG
WAKY56R	CCAAATTGGACAGCTGACAG
MYB305F	TGGAGAAAGGGACCATGGAC
MYB305R	CCTTTTCAACCCTGTGAGCC
MYCF	CCAAATCCTTGTTCTCCTCG
MYCR	CTTCCCGCTGAGATCATTTC
Laccase9F	GAATCAGGGAGAATATGGCG
Laccase9R	TCGGACACTGCGTAATGTTC
Laccase12F	CAGACCACGGATGTCCTGATC
Laccase12R	GGTGGTGTTGTCAAATGGGG
Laccase14F	AGAACATCACGCAGTGCCCC
Laccase14R	GCGTGCCACCAAAGAGTTCC
Peroxidase11F	CTCCAAGTTCCTCTATCAGGGC
Peroxidase11R	AAGTCTTCGTAGATCCGCGC
Peroxidase15F	TCCAGGGATGTGATGGGTCG
Peroxidase15R	CGGCCTTCATGTCATCGACC
Peroxidase64F	AGAGCTGTAGCACTAGCAGTGTTC
Peroxidase64R	CATTTGTGACCAGAGACTCGAG

## Abbreviations

PAL: Phenylalanine ammonialyase
C4H: Cinnamic acid 4-hydroxylase
C3H: Coumarate 3-hydroxylase
4CL: 4-coumarate coenzyme A ligase
CCR: Cinnamoyl-CoA reductase
CAD: Cinnamyl alcohol dehydrogenase
COMT: Caffeic acid-O-methyltransferase
CCoA-OMT: Caffeoyl-CoA O-methyltransferase
F5H: Ferulate-5-hydroxylase
ORF: open read fragment
DAB: Days after bloom
bp: base pair
cDNA: complementary DNA
qRT-PCR: quantitative real time polymerase chain reaction
GO: Gene Ontology
DEG: differential expression gene
RPKM: Reads Per Kilobase of exon model per Million mapped reads
bHLH: basic helix–loop-helix proteins
